# Warm Ambient Temperature Decreases Food Intake in a Simulated Office Setting: A Pilot Randomized Controlled Trial

**DOI:** 10.3389/fnut.2015.00020

**Published:** 2015-08-24

**Authors:** Molly C. Bernhard, Peng Li, David B. Allison, Julia M. Gohlke

**Affiliations:** ^1^Department of Environmental Health Sciences, School of Public Health, University of Alabama at Birmingham, Birmingham, AL, USA; ^2^Office of Energetics and Nutrition Obesity Research Center, University of Alabama at Birmingham, Birmingham, AL, USA

**Keywords:** obesity, thermal environment, thermoneutral zone, food intake, heat dissipation

## Abstract

**Background:**

We hypothesized that exposure to temperatures above the thermoneutral zone (TNZ) would decrease food intake in young adults in a sedentary office environment over a 2-h period.

**Methods:**

Participants wearing standardized clothing were randomized to perform routine office work in the TNZ, considered control (19–20°C), or above the TNZ considered warmer (26–27°C) using a parallel-group design (*n* = 11 and 9, respectively). Thermal images of the inner canthus of their eye and middle finger nail bed, representing proxies of core and peripheral temperatures, respectively, were taken at baseline, first, and second hour during this lunchtime study. Heat dissipation was estimated using peripheral temperature. General linear models were built to examine the effects of thermal treatment on caloric intake and potential mediation by heat dissipation. Researchers conducted the trial registered as NCT02386891 at Clinicaltrials.gov during April to May 2014.

**Results:**

During the 2-h stay in different ambient temperatures, the participants in the control conditions ate 99.5 kcal more than those in the warmer conditions; however, the difference was not statistically significant. Female participants ate about 350 kcal less than the male participants (*p* = 0.024) in both groups and there was no significant association between caloric intake and participant’s body mass index (BMI). After controlling for thermal treatment, gender and BMI, the participant’s peripheral temperature was significantly associated with caloric intake (*p* = 0.002), suggesting a mediating effect. Specifically, for every 1°C increase in peripheral temperature suggesting increased heat dissipation, participants ate 85.9 kcal less food.

**Conclusion:**

This pilot study provided preliminary evidence of effects of thermal environment on food intake. It suggests that decreased food intake in the experimental (warmer) environment is potentially mediated through thermoregulatory mechanisms.

## Introduction

Previous studies suggest increased time spent outdoors is associated with lower body mass index (BMI) (1, 2). Increased usage of central heating and air conditioning systems coupled with increased time spent indoors has created an environment where humans are presumably expending less energy to regulate their body temperature (3). The range of ambient temperatures with which a healthy adult can maintain body temperature without expending energy beyond the normal basal metabolic rate is the thermoneutral zone (TNZ) (4). Outside of the TNZ, the body must adapt through thermoregulation to maintain body temperature (4). Energy intake and expenditure are adjusted at temperatures above and below the TNZ (4, 5).

Research in mice and livestock shows that food intake and subsequently, weight gain is reduced in high-ambient temperatures (6–9). In addition in young, physically active, healthy men, food intake is inversely proportional to temperatures above the TNZ (10). Yet, there is minimal information on how small changes in ambient temperature affect food intake in the general public. In particular, there is limited information on how small changes in ambient temperature exposure above the TNZ affect food intake. Recent research suggests that small changes in diet and physical activity over time could lead to prevention of further weight gain (11). One approach may be altering the ambient temperature to lead to changes in consumption practices.

This pilot study tested the study procedures and measurement techniques, estimated the feasibility of recruitment, and estimated variance related to the outcome measures (12). We hypothesized that ambient temperature above the TNZ decreases the food intake of sedentary young people in an office situation. To conduct this pilot study, we used a randomized controlled trial design. Participants were randomized to perform office work in a simulated office setting at thermoneutral (control) or warmer temperatures. To estimate parameter variance for future studies, we estimated changes in thermoregulation, determined differences in food intake by thermal condition, and assessed if differences in thermoregulation between the two temperature conditions mediate food intake in the two thermal conditions.

## Materials and Methods

### Trial design and overview

A parallel-group trial design was utilized where approximately half of participants would receive each treatment. Researchers conducted the trial (Registered as NCT02386891 at Clinicaltrials.gov) from April to May 2014 according to the trial protocols as approved by the UAB Institutional Review Board (IRB protocol #X140206006). Young adults were randomly assigned to a thermoneutral or warmer condition to perform office work for 1 h. The second hour they ate lunch and were able to continue their work when ready. Following this, body weight and height were measured. The total exposure time was 2 h.

### Participants

Due to a lack of sufficient evidence in the current literature to calculate the sample size needed to see a significant difference between groups, participants (*n* = 20) were included based on feasibility during April–May 2014. Participants were recruited from the downtown Birmingham campus of the University of Alabama at Birmingham (UAB) via flyers in common spaces (parking decks, libraries, academic classrooms, greenspaces, and communal eating areas). Subjects were screened for initial eligibility via telephone questionnaire (Data sheet S1 in Supplementary Material). Subjects with ages of 19–35 years for both genders and all races and ethnicities were eligible. Prospective participants were excluded based on self-report of major medical condition, medication that may affect their heat tolerance, blood pressure or appetite, histories of eating disorders, smoking, pregnancy or <3 months post-partum, recent weight loss or gain >5% of body weight in the last 6 months, food allergies or dietary restrictions, and anyone with a potential conflict of interest. After trial commencement, no changes were made to the protocol.

### Randomization

During the screening phone call, participants were given limited information about the purpose of the study so as not to influence their behavior during the study. The participant was told that researchers were interested in the effects of thermal environment on routine office work in the range of temperatures (19–27°C, 66–81°F), and they would sit in an enclosed office during late morning (10:30 a.m.) performing sedentary tasks they brought with them – reading, writing, or working on a laptop on the provided desk. They were fully informed of the goals of the study during de-briefing at the end of their participation. Researchers generated the randomization, enrolled participants, and assigned participants to interventions. Researchers were aware of the randomization in order to adjust the thermal environment to achieve the desired temperature range, but did not reveal to the participants. After confirming their scheduled visit the day before participation, participants were randomized to either a warmer 26–27°C (79–81°F) or a control 19–20°C (66–68°F) environment, using the website http://www.random.org/ to generate random numbers of 1 (control environment) or 2 (warm environment). No blocking was done to ensure equal groups.

### Experimental procedures

Participants went through the same protocols at the same time on each designated appointment with the sole difference being their randomization to thermal environment. Thermal environments were controlled using the thermostat and a space heater and verified by HOBO^®^ Pendant temperature/light data loggers (Onset Corp. UA-002-64) (two in the simulated office, and one outside of the building) taking temperature measurements every 1 min. Bernhard et al. previously reported based on manufacturer specifications, monitors can detect temperatures ranging from −20 to 70°C with accuracy of 0.47°C and resolution of 0.10°C at 25°C (13). Four monitors in the same controlled temperature indoor location over a 5-day period gave an average SD between monitor readings of 0.04°C (13). At the scheduled visit, participants went through formal consent process one-on-one with personnel and they completed a demographic questionnaire. A baseline thermal image was taken. They wore standardized clothing (long pants, closed-toed shoes, socks, and the provided cotton short-sleeve t-shirt) and were encouraged not to leave the room. If they needed to use the restroom, they were escorted to the restroom and the time was recorded.

After 1 h (midpoint), trained personnel entered the room and took a second thermal image capturing estimated core and peripheral temperature at the midpoint. After the thermal image, they presented the participant with lunch at 11:30 a.m. – one large, cheese pizza from a national restaurant chain, plate, utensils, napkins, and water. This test meal was provided in excess so that each participant had leftover pizza. The pizza was cut into a grid, not the typical slices. Participants were instructed to eat/drink at their leisure and then continue working on their office tasks.

At the end of the second hour (end point), staff entered the room, and took a third thermal image of core and peripheral temperatures. They informed the participant that the emphasis of the study was on quantity of food consumed and fully debriefed each on the details of the study that the food waste will be weighed. The participant was escorted to a body composition laboratory where weight and height were measured on a calibrated scale and stadiometer, respectively. BMI was calculated as kg/m^2^. All testing was performed by trained personnel, and all equipment was calibrated daily before testing.

The primary outcome of energy intake was measured by weighing the remaining food and comparing to known caloric content. The caloric content of food provided was verified through bomb calorimetry (14) of an average of three samples by the UAB Nutrition Obesity Research Center core laboratory.

### Infrared image

Trained staff used an infrared thermal camera (FLIR T300) to capture an estimate of subject’s core and peripheral temperature from the inner canthus of the eye and third nail bed, respectively, after randomization (15). This served as a biomarker of exposure to quantify estimated changes in thermoregulation. Thermal images were taken according to the protocols of Savastano et al. (16). Participants were clothed and remained in a sedentary state in the environment for 15 min before the first image. Participants were instructed not to touch hands to another body part or the table for 5 min before the image to prevent heat sharing. A trained staff member stood 3 feet in front of the participant, who was standing against a wall. Taking thermographic photos 15 min after entering the room (labeled hereafter as baseline), after 1 h of exposure (midpoint), and at the end of the second hour of exposure serve as an indication of whether increased/decreased skin blood flow to the periphery was initiated to dissipate heat in the body. For all models in this analysis, the midpoint measure was used. Each participant answered an oral questionnaire regarding factors that could influence their thermoregulation like food or drink intake that morning, caffeine intake, physical exertion, or hot showers.

### Statistical analysis

The baseline and post-exposure characteristics between groups were compared by *t*-test for continuous variables or Fisher’s exact test for categorical variables. The effects of ambient temperature on caloric intake was examined using a linear regression model after controlling for participants’ gender and BMI. A further exploratory analysis was conducted to examine the association between caloric intake and the peripheral temperature estimates, using a linear regression model after controlling for thermal treatment, participants’ gender and BMI. All analyses were conducted using SAS 9.4 (Cary, NC, USA).

## Results

A participant flow diagram shows the number of individuals recruited, eligible, allocated to treatment, and included in analyses (Figure S1 and Data sheet S2 in Supplementary Material). There was no harm to participants and no measured unintended effects in either treatment arm. The trial ended when participants completed the protocols (*n* = 20).

### Demographic and thermal questionnaire

There were no significant differences in the demographics between the thermal treatments (Table 1). All participants reported having air conditioning in their home and spending two or more hours per day in a location other than their home with air conditioning. All participants reported being non-smokers. Fifteen participants reported eating 3 h prior to participation. Sixteen reported having a drink, of those six were caffeinated and five were hot beverages. Six participants had taken a hot shower 3 h or less prior to participation, but none reported physically exerting themselves. Five participants reported wearing either nail polish or acrylic nails and nine reported wearing topical creams or makeup on their hand or eye areas. The majority of participants reported being single (*n* = 17).

**Table 1:** **Summary of participant demographics by thermal condition**.

	(19–20°C)	(26–27°C)	*p* values
***N***	11	9	–
**Female (%)**	8 (72.7)	5 (55.6)	0.64
**Age (SD)**	23.1 (4.0)	23.2 (2.0)	0.93
**Race**			0.20
Black or African-American (%)	6 (54.5)	2 (22.2)	
Other (%)	5 (45.5)	7 (77.8)	
**Employment/student status**			1.0
Student and/or student + employed (%)	10 (90.9)	8 (88.9)	
Employed only (%)	1 (9.1)	1 (11.1)	
**Income**			1.0
<$20,000 or unknown (%)	3 (27.2)	2 (22.2)	
$20,000–$49,999 (%)	8 (72.8)	7 (77.8)	
**Body mass index**			
BMI (SD)	27.5 ± 6.0	24.3 ± 4.0	0.19
Normal weight (*n*)	4	5	
Overweight (*n*)	3	4	
Obese (*n*)	4	0	

### Thermal images

**Figure 1:**
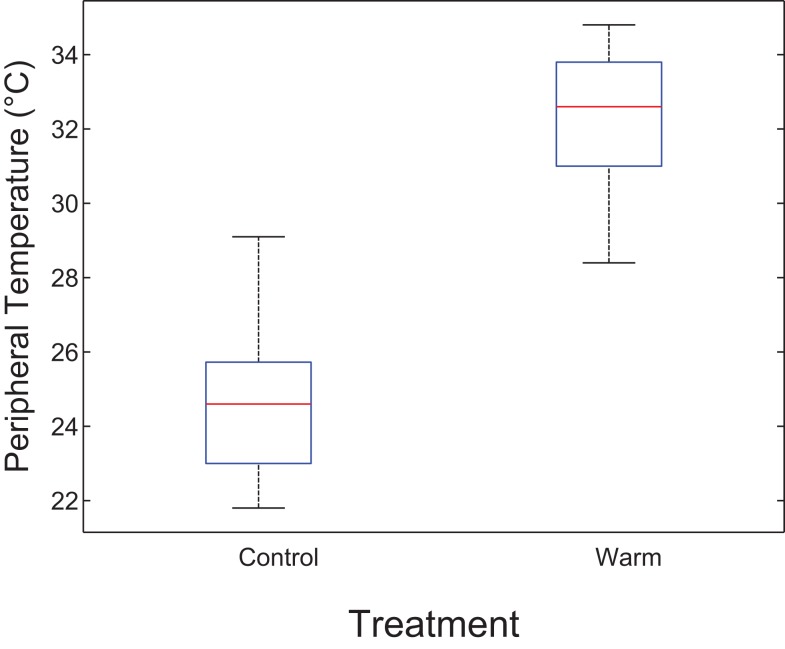
**Boxplot indicating differences in estimated heat dissipation at midpoint by thermal environment**. The central mark is the median and the edges are the 25th and 75th percentiles. The whiskers are the most extreme data points. After 1 h (at midpoint prior to food intake), peripheral temperature in the control group was significantly lower than in the warmer group (24.8 vs. 32.2, *p* < 0.0001). This suggests reduced heat dissipation in the control group.

Estimates of core temperatures measured from a thermal image of the inner canthus of the eye varied from the standard 37°C (Table S1 in Supplementary Material). Peripheral temperatures from thermal images were used to estimate heat dissipation to the extremities. After 1 h (at the midpoint of the thermal treatment and prior to food intake), the average peripheral temperature in the control group was significantly lower than in the warmer group (24.8 vs. 32.2, *p* < 0.0001) (Figure 1).

### Energy intake

During the 2-h stay in different ambient temperatures, the participants in the control condition ate more than those in the warmer condition. Adjusting for BMI and gender, the caloric intake difference was about 99.5 kcal between the participants in two different temperatures; however, the difference was not statistically significant (Table 2). Female participants ate 352.0 kcal less than the male participants (*p* = 0.024) in both groups. There was no significant association between caloric intake and participant’s BMI.

**Table 2:** **The effect of thermal treatment on food intake (kcal)**.

Parameter	Estimate	95% Confidence intervals	*p* Values
Intercept	662.3	(−55.9, 1380.4)	0.071
Control treatment (19–20°C)	99.5	(−196.5, 395.5)	0.510
Female	−352.0	(−657.2, −46.9)	0.024
BMI	15.7	(−13.4, 44.7)	0.290

**Figure 2:**
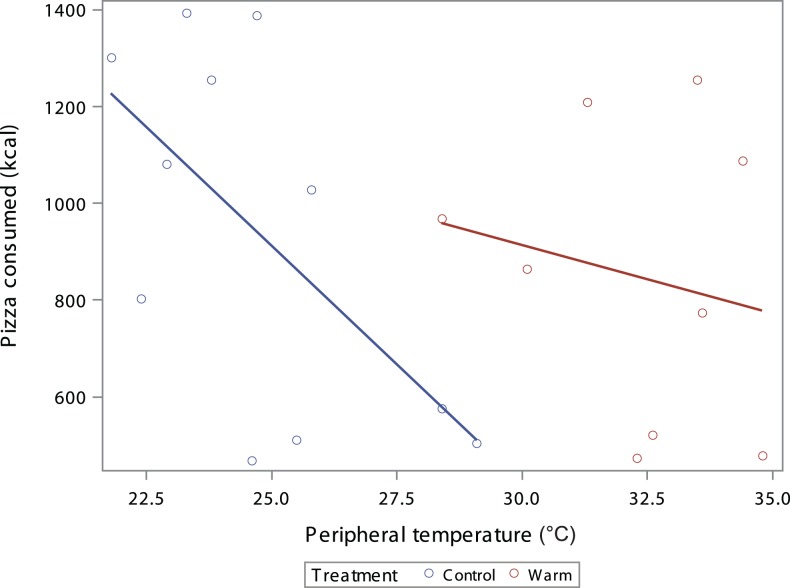
**Scatterplot of the kcal consumed by participants peripheral temperature °C with dots as individual participants and lines as best fit by treatment; coded by color (red = warm treatment, blue = control treatment)**. There is a negative correlation between food intake and the estimate of peripheral temperature (Control *R*^2^ = 0.37, *p* = 0.046, Warm *R*^2^ = 0.037, *p* = 0.62).

There was a negative correlation between food intake and peripheral temperature as measured by thermal images (Figure 2). However, the negative trend was only significant in the control group (*p* = 0.046) and not in the warm group (*p* = 0.62) likely due to small sample size. This supports the hypothesis that reduced heat dissipation during the treatment is associated with greater food intake.

To explore whether the difference in food intake by thermal treatment was mediated by reduced heat dissipation, a second linear model was created. The thermal treatment, gender and BMI adjusted association between caloric intake and heat dissipation was examined and the results are shown in Table 3. After controlling for thermal treatment, gender and BMI, the participant’s peripheral temperature was significantly associated with caloric intake (*p* < 0.001), suggesting a mediating effect (17). Specifically, for every 1°C increase in peripheral temperature indicating increased heat dissipation, participants ate 85.9 kcal less food. Gender and BMI were also significant predictors in this model.

**Table 3:** **Regression model of food intake including peripheral temperature from thermal image taken after 1 h of treatment (prior to food presentation)**.

Parameter	Estimate	95% Confidence intervals	*p* Values
Intercept	3188.8	(1437.8, 4939.7)	<0.001
Average peripheral temperature (°C)	−85.9	(−142.1, −29.8)	0.002
Control treatment (19–20°C)	−583.4	(−1090.7, −76.2)	0.02
Female	−365.9	(−615.2, −116.7)	0.004
BMI	26.3	(1.6, 51.0)	0.03

## Discussion

Previous studies have suggested that increases in obesity may be related to increased time spent in the TNZ (18–20). This study sought to better understand how thermal environment affects acute food intake. One may speculate that both the acute thermogenic effect of food intake and long-term increased fat storage in a cold environment may be advantageous for retaining heat (21). Other forms of thermoregulation, such as blood flow, are initiated centrally in the hypothalamus, which is also an area of the CNS known to regulate food intake (22). The differences in food intake by thermal environment found in this pilot study are in the same direction as those found in Westerterp-Plantega et al. (23). They found that women did not report thermal discomfort at 27°C, but did significantly decrease their energy intake in the short-term relative to 22°C and that the decrease in energy intake was related to an increase in skin temperature (23). Results from the present study also suggest reduced heat dissipation (as measured by estimated peripheral temperatures from a thermal image) may mediate the relationship between food intake and thermal environment.

This present study is limited primarily by small sample size and differences in response between sexes. Participants may have been distracted while eating with the potential for higher energy intake (24) or restricted their intake in the new setting as seen in some laboratory studies (25). In addition, food intake following exposure was not measured; therefore, we are unable to account for potential compensatory food intake following the exposure. A minor limitation is a lack of a true intent to treat analysis. Four participants were eligible via the phone screening and scheduled to participate, researchers randomized the participants after confirming their appointment the day prior to their participation to prepare the room conditions. The participants were blinded to their randomization and did not show up (*n* = 3) or canceled due to inclement weather (*n* = 1) (Figure S1 and Data sheet S2 in Supplementary Material). Thus, it is unlikely that their lack of participation is related to their randomization, but a true intent to treat analysis was not possible. Further examination of the utility of thermal images for estimating relative heat dissipation, and in particular the use of the inner canthus of the eye as an adequate proxy for core temperature across body types is needed based on the variation we detected in our sample (32.6–37.2°C) and the limitations of alternative methods of detection (26, 27). Investigators of future studies may want to consider having participants fast 8 h prior to the start of the study, limit strenuous physical activity at least 48 h prior, increase exposure time, further limit the age of participants (28), control for the effect of menstrual cycle stage (29), screen participants for dietary restraint (30), incorporate a cross-over design, and include work performance measures. In addition, the control group temperatures were at the lower end of the range of temperatures for the TNZ. The human TNZ is varied within and between individuals, based on age, sex, disease state, and fat distribution among others.

In conclusion, this pilot study suggests that acute food intake may be reduced in warmer environments and informs study design issues that will be helpful in implementation of larger studies designed to test the effectiveness of altering ambient temperatures to affect food intake.

## Author Contributions

MB, PL, DA, and JG had substantial contributions to the design of the work, analysis, and interpretation for the work. All authors were involved in drafting and critically revising the work and approval of the final document submitted. All authors are accountable for investigating and resolving any questions related to the accuracy or integrity of the work.

## Conflict of Interest Statement

The authors declare that the research was conducted in the absence of any commercial or financial relationships that could be construed as a potential conflict of interest.

## Supplementary Material

The Supplementary Material for this article can be found online at http://journal.frontiersin.org/article/10.3389/fnut.2015.00020
