# Corrigendum: Warm Ambient Temperature Decreases Food Intake in a Simulated Office Setting: A Pilot Randomized Controlled Trial

**DOI:** 10.3389/fnut.2015.00036

**Published:** 2015-11-04

**Authors:** Molly C. Bernhard, Peng Li, David B. Allison, Julia M. Gohlke

**Affiliations:** ^1^Department of Environmental Health Sciences, University of Alabama at Birmingham, Birmingham, AL, USA; ^2^Office of Energetics and Nutrition Obesity Research Center, University of Alabama at Birmingham, Birmingham, AL, USA; ^3^Department of Population Health Sciences, Virginia-Maryland College of Veterinary Medicine, Blacksburg, VA, USA

**Keywords:** obesity, thermal environment, thermoneutral zone, food intake, heat dissipation

Page 3 of the article by Bernhard et al. (2015) contained a minor error, which we hereby correct. There were two separate sources that informed a statement within the text on page 3 “Trained staff used an infrared thermal camera (FLIR T300) to capture an estimate of subject’s core and peripheral temperature from the inner canthus of the eye and third nail bed, respectively after randomization.” They were mistakenly formatted as one source originally listed as Bach AJ, Stewart IB, Disher AE, Costello JT. Comparison of measuring sites for the assessment of body temperature. Thermol Int (2009) 19(2):35–42. doi:10.1371/journal.pone.0117907

The two sources should have been listed separately as follows:

Bach AJ, Stewart IB, Disher AE, Costello JT. A comparison between conductive and infrared devices for measuring mean skin temperature at rest, during exercise in the heat, and recovery. *PLOS One* (2015) **10**(2):e0117907. doi:10.1371/journal.pone.0117907

Pascoe DD, Fisher G. Comparison of measuring sites for the assessment of body temperature. *Thermol Int* (2009) **19**(2): 35–42.

This error does not change the scientific conclusions of the article in any way.

## Author Contributions

MB and JG drafted this corrigendum. All authors have read and approved this corrigendum. All authors are accountable for investigating and resolving any questions related to the accuracy or integrity of the work.

## Conflict of Interest Statement

The authors declare that the research was conducted in the absence of any commercial or financial relationships that could be construed as a potential conflict of interest.

