# Supplementary material for: Warm Ambient Temperature Decreases Food Intake in a Simulated Office Setting: A Pilot Randomized Controlled Trial
Source: Front Nutr. 2015 Aug 24;2:20. doi: 10.3389/fnut.2015.00020 (PMC4500895; doi:10.3389/fnut.2015.00020)
Supplement: Supplementary file 1 [file data_sheet_1.docx]

**Screening Questionnaire:**

**Potential participants will be called between 8am and 8pm to screen for appropriate participants. The call is intended to ask potential participants questions to determine eligibility of participating in the study, interest in participating in the study, and scheduling a time to participate in the study.**

Hello, this is ___________ from the University of Alabama at Birmingham. Could I speak with _________________?

Hello ___________, this is ___________ from the University of Alabama at Birmingham. I’m calling because you expressed interest in participating in a study at the University of Alabama at Birmingham. Is now a good time to talk about this or should I call back at another time? If so, when?

*If this is a good time, proceed. If not, ask for a better time to talk and say that you will call back then.

Would it be alright if I told you some information about the study and then asked you some questions to see if you are eligible to participate?

After this initial phone screening, participants will be asked to come to the Ryals School of Public Health (Ryals) building, Suite 530. Parking will be available outside Ryals at the 9^th^ Avenue parking deck on the 1^st^ floor for a fee or they may park in their regular parking spot and walk or take the UAB bus.

After going through the formal consent process one-on-one with personnel, you will be randomized to either a warmer (75°F) or a colder (65°F) environment. We are not able to divulge all of the details of the study until after the 2 hour period as we do not want to influence their behavior. We will NOT use video monitoring. If you are uncomfortable you may choose not to participate or leave at that time or at any time during the study.
You will be asked to wear long pants and closed-toed shoes. That day you will be presented with a t-shirt and asked to go to the restroom to change your top. At this time, you will be instructed that you will be asked to spend approximately 2 hours in a room with the door closed and this is a good opportunity to use the restroom. Trained staff will use an infrared thermal camera (FLIR T300) to capture your core and peripheral temperature, the inner canthus of the eye and third nail bed respectively, prior to entering the thermal environment. Researchers are interested in the effects of thermal environment on routine office work. You will sit in an enclosed office (the thermal environment) performing typical, sedentary office tasks like reading a passage and answering questions for one hour. You will be instructed to keep the door closed and perform tasks. You have already been encouraged to use the restroom prior to starting the time in the office. However, if you need to use the restroom, you will be escorted to the restroom and time will be recorded.

After one hour, trained personnel will enter the room and take a second thermal image capturing core and peripheral temperature. They will also present you with a large, cheese Mellow Mushroom pizza, plate, utensils, napkins, and water for your lunch. You can eat/ drink at your leisure and then continue working on your office tasks.
At the end of the second hour, staff will describe the details of the study. Researchers will take you to the Webb building room 204/205 to be weighed and have your height measured.

Are you still interested in participating?
*If no longer interested in participating thank the individual for his or her time.

Can I ask you a few questions to see if you are eligible to participate in the study?

Are you a man or woman?
What is your age? Are you between the ages of 19 to 35?
Do you have any food allergy, including, but not limited to lactose or gluten intolerance?
Do you have any religious affiliations that include specific food guidelines?
Do you have any personal dietary restrictions, including but not limited to veganism?
Have you participated in any weight-reduction program, weight-loss diet, or other special diet within the previous 3 months?
Have you had weight loss or gain of >5% of body weight in the past 6 months for any reason except post-partum weight loss?
Are you currently taking medication that suppresses or stimulates appetite or that affects body weight, including oral anti-diabetics medications?
Do you have a history of prior surgical procedure for weight control or liposuction?
Are you currently taking statins?
Do you have high blood pressure (above 140 systolic, 90 diastolic)?
Are you a current smoker or have you quit smoking less than 6 months prior?
Do you have any major disease, including:

- Active cancer or cancer requiring treatment in the past 2 years (except nonmelanoma skin cancer).
  - Active or chronic infections, including self-reported HIV positivity and active tuberculosis.
  - Active cardiovascular disease or event including hospitalization or therapeutic procedures for treatment of heart disease (e.g., coronary artery bypass, percutaneous transluminal coronary angioplasty) in the past 6 months; New York Heart Association Functional Class >2 with respect to congestive heart failure; stroke or transient ischemic attack in the past 6 months.
  - Gastrointestinal disease, including self-reported chronic hepatitis or cirrhosis, any episode of alcoholic hepatitis or alcoholic pancreatitis within past year, inflammatory bowel disease requiring treatment in the past year, recent or significant abdominal surgery (e.g., gastrectomy).
  - Active renal disease.
  - Lung disease: chronic obstructive airway disease requiring use of oxygen.
  - Diagnosed diabetes (type 1 or 2).
  - Uncompensated or uncontrolled psychiatric disease (such as schizophrenia and bipolar disorder) that, in opinion of the investigators, would impede conduct of the trial or completion of procedures.

Do you have a history of or currently have an eating disorder?
Are you willing and able to give informed consent?
Are you able to communicate with the pertinent staff?
Do you have another household member is a participant or staff member in the trial?
Are you currently or do you anticipate participating in another intervention research project that would interfere with the intervention offered in the session?
Are you willing to accept condition that you are randomized to?
Are you currently taking antidepressant, steroid, or thyroid medication, unless dosage has been stable for at least 6 months?
Do you have a recent or ongoing problem with drug abuse or addiction?
Do you on average consume 3 or more alcohol containing beverages daily?
Have you consumed 7 or more alcoholic beverages within a 24-hr period in the past 12 months?
Are you a student in the Environmental Health Science Department, Nutrition Sciences School, any student, trainee, or post-doctoral fellow who is directly or indirectly receiving funding from the Nutrition Obesity Research Center?
Are you comfortable reading independently and answering a couple written questions related to the passage that you’ve read?
Are you willing to sit in a small office with a closed door for two hours?
Do you feel uncomfortable in confined spaces for extended periods of time?
Are you willing to have your height and weight taken?

For females only:
Are you currently pregnant or less than 3 months post-partum?
Are you currently nursing or within 6 weeks of having completed nursing?
Do you anticipate a pregnancy between time of screening and session date?
Are you willing to report possible or confirmed pregnancies promptly at the time of the session?

If eligible, (schedule a time for the participant to come in for the study).
If ineligible, thank the individual for his or her time.

Thank you for your time. Please give us or UAB researchers a call at any time if you have any questions. You can reach me at 205-567-1858 or Julia Gohlke, the UAB study investigator at 205-934-7060.
