# Supplementary figures and images for: Warm Ambient Temperature Decreases Food Intake in a Simulated Office Setting: A Pilot Randomized Controlled Trial
Source: Front Nutr. 2015 Aug 24;2:20. doi: 10.3389/fnut.2015.00020 (PMC4500895; doi:10.3389/fnut.2015.00020)

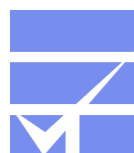

# CONSORT

TRANSPARENT REPORTING of TRIALS

## CONSORT 2010 Flow Diagram

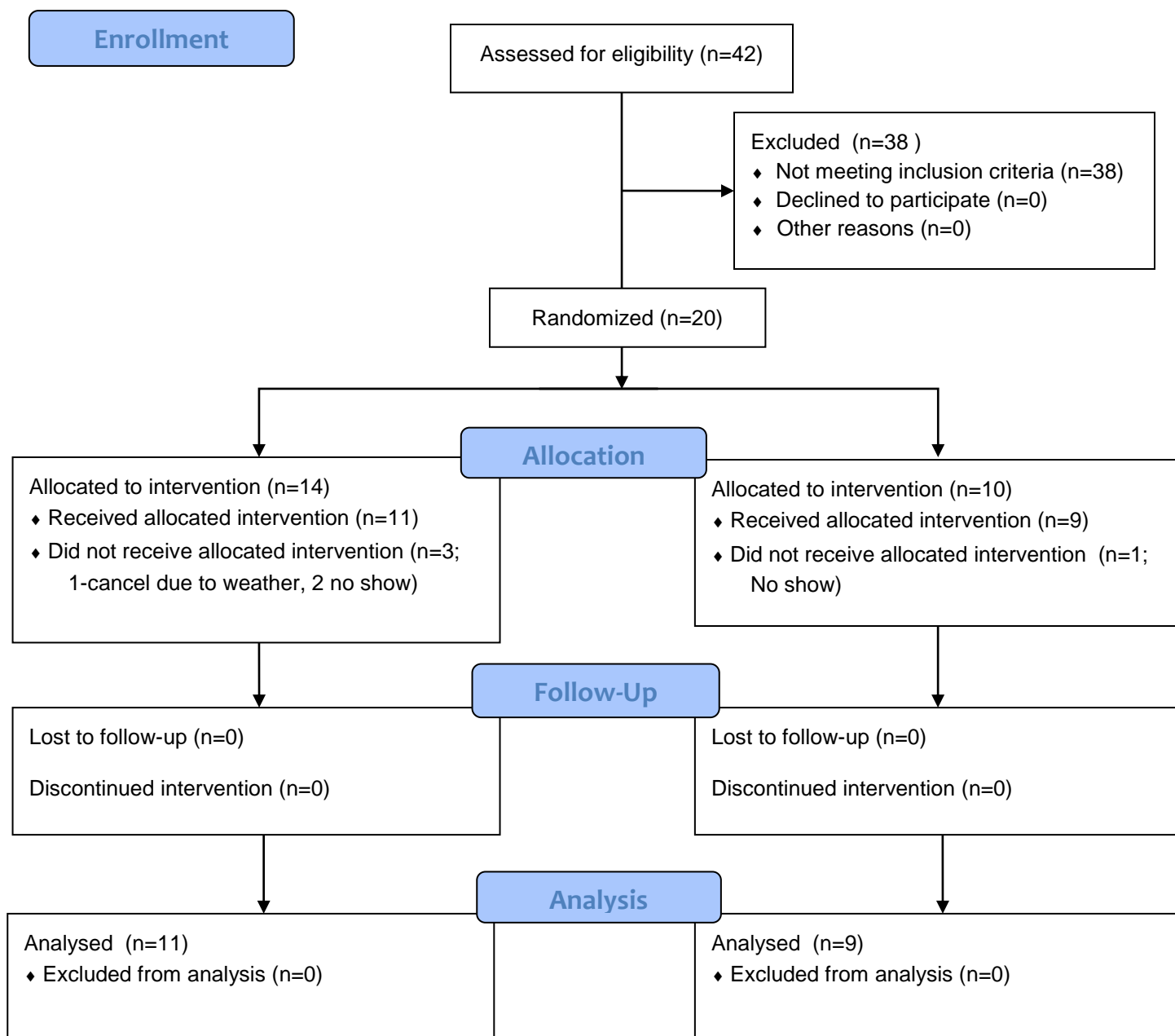

Supplement: Supplementary file 3 [file image_1.pdf]
