# Supplementary material for: Warm Ambient Temperature Decreases Food Intake in a Simulated Office Setting: A Pilot Randomized Controlled Trial
Source: Front Nutr. 2015 Aug 24;2:20. doi: 10.3389/fnut.2015.00020 (PMC4500895; doi:10.3389/fnut.2015.00020)
Supplement: Supplementary file 4 [file table_1.docx]

Supplemental Table 1: Thermal image estimates of core (inner canthus of eye) and peripheral (3 finger nail bed) temperatures prior to treatment (baseline), 1 hr. after start of treatment (midpoint), and at the end of treatment (2 hrs.) after eating lunch.

|  |  | Control Treatment | | Warm Treatment | |
| --- | --- | --- | --- | --- | --- |
|  |  | Core Temperature (°C) | Peripheral Temperature (°C) | Core Temperature (°C) | Peripheral Temperature (°C) |
| Baseline | Average (SD) | 33.3 (0.8) | 29.5 (3.4) | 35.2 (0.7) | 34.3 (0.6) |
|  | Range | 32.6, 35.4 | 22.5, 34 | 34.6, 36.5 | 33, 34.9 |
| Midpoint | Average (SD) | 33.9 (1.2) | 24.8 (2.3) | 35.1 (0.5) | 32.3 (2.1) |
|  | Range | 32.6, 37.2 | 21.8, 29.1 | 34.2, 35.7 | 28.4, 34.8 |
| Final | Average (SD) | 34.0 (1.0) | 23.8 (1.3) | 34.9 (0.4) | 31.6 (2.0) |
|  | Range | 32.8, 35.9 | 22.1, 25.9 | 34.2, 35.4 | 28.7, 34.2 |
